# Mini-review: Mitochondrial DNA methylation in type 2 diabetes and obesity

**DOI:** 10.3389/fendo.2022.968268

**Published:** 2022-08-25

**Authors:** Emma K. Rautenberg, Yassin Hamzaoui, Dawn K. Coletta

**Affiliations:** ^1^ Department of Physiology, The University of Arizona College of Medicine, Tucson, AZ, United States; ^2^ Department of Medicine, Division of Endocrinology, The University of Arizona College of Medicine, Tucson, AZ, United States; ^3^ Center for Disparities in Diabetes, Obesity and Metabolism, The University of Arizona, Tucson, AZ, United States

**Keywords:** mitochondria, epigenetics, methylation, insulin resistance, skeletal muscle, obesity

## Abstract

Type 2 diabetes (T2D) and obesity are two of the most challenging public health problems of our time. Therefore, understanding the molecular mechanisms that contribute to these complex metabolic disorders is essential. An underlying pathophysiological condition of T2D and obesity is insulin resistance (IR), a reduced biological response to insulin in peripheral tissues such as the liver, adipose tissue, and skeletal muscle. Many factors contribute to IR, including lifestyle variables such as a high-fat diet and physical inactivity, genetics, and impaired mitochondrial function. It is well established that impaired mitochondria structure and function occur in insulin-resistant skeletal muscle volunteers with T2D or obesity. Therefore, it could be hypothesized that the mitochondrial abnormalities are due to epigenetic regulation of mitochondrial and nuclear-encoded genes that code for mitochondrial structure and function. In this review, we describe the normal function and structure of mitochondria and highlight some of the key studies that demonstrate mitochondrial abnormalities in skeletal muscle of volunteers with T2D and obesity. Additionally, we describe epigenetic modifications in the context of IR and mitochondrial abnormalities, emphasizing mitochondria DNA (mtDNA) methylation, an emerging area of research.

## Introduction

### Type 2 diabetes and obesity: Insulin resistance

Approximately 11.3% of the United States population has diabetes, with the majority represented by type 2 diabetes (T2D). Many patients with T2D are obese, with up to 85% in some populations (1). In addition, obesity cases have reached epidemic proportions, with more than two-thirds of American adults being overweight or obese (2). T2D and obesity are two of the most challenging public health problems of our time, and treating these diseases costs billions of dollars a year in the United States (3, 4). The total cost of diagnosed diabetes rose from $245 billion in 2012 to $327 billion in 2017, a 26% increase over 5 years (5). In 2016, the medical expenditure cost of obesity in adults was $260.6 billion in the United States (6). Additionally, obese adults have annual medical expenses $2,505 higher than people in a healthy weight category (6). The economic burden is at an all-time high, and it is crucial to research and identify the cellular, biochemical and molecular abnormalities that contribute to the pathophysiology of T2D and obesity. One pathophysiological condition that underlies T2D and obesity is insulin resistance (7).

Insulin resistance is a reduced response of the skeletal muscle, white adipose tissue, and liver to the biological effect of insulin (7–10). In normal physiological conditions, insulin enables cells to take glucose from the bloodstream to use it for energy and storage (9, 10). When blood glucose levels rise, which typically occurs after a meal, the β cell of the pancreas release insulin to keep blood glucose within normal homeostatic ranges. When insulin resistance develops, the pancreas produces more insulin, termed hyperinsulinemia, to keep blood glucose levels within the normal range (7–9). Prediabetes occurs when the pancreas can no longer maintain a state of hyperinsulinemia to keep blood glucose levels within normal homeostatic levels. Finally, if the insulin resistance worsens and beta-cell dysfunction occurs, T2D ensues (8, 11).

Insulin resistance at peripheral tissues results in decreased insulin-stimulated glucose uptake in skeletal muscle and white adipocytes, and impaired suppression of liver glucose production (9, 11). Many factors contribute to whole-body insulin resistance, including lifestyle variables such as a high-fat diet and physical inactivity resulting in obesity (12–14), age (15), genetics (16), low-grade inflammation (17), and impaired mitochondrial function (18–20). The mitochondrial abnormalities observed in insulin-resistant skeletal muscle are notable since this tissue is the main site of insulin-stimulated glucose uptake (21) and thus consequential to glucose dysregulation, as occurs in prediabetes and T2D. The next section of this mini-review will describe the normal function and structure of mitochondria and proceed with highlighting some of the studies that highlight mitochondrial abnormalities in skeletal muscle of volunteers with T2D and obesity.

### Mitochondria: Structure, function, genome and transcription

Mitochondria are double-membrane organelles found in almost all eukaryotic cells. They engage in several functions in the cell, including regulation of calcium homeostasis (22), redox status (23), cell growth and death (24), and adenosine triphosphate (ATP) production (25). Mitochondria provide over 90% of ATP required for cell metabolism, with ATP production starting when pyruvate from cytosolic glycolysis enters the Krebs cycle in the mitochondrion’s matrix (26). The NADH, FADH, and GTP produced in the Krebs cycle power the electron transport chain (ETC) found in the mitochondria’s inner membrane, and the proteins of the ETC allow a series of oxidation-reduction reactions to occur (25, 26). This produces a proton gradient across the inner membrane that drives the phosphorylation of adenosine diphosphate (ADP) to form ATP *via* ATP synthase (25, 26). This process is known as chemiosmotic coupling of oxidative phosphorylation (27).

Mitochondria contain their own DNA, called mitochondrial DNA (mtDNA) (28). Ninety-nine percent of mtDNA is inherited solely from the mother, and mitochondria replicate and perform apoptosis separately from the other cell organelles (29). The mtDNA is 16,569 base pairs organized as circular, covalently closed, double-stranded DNA (28). The two strands are differentiated by their nucleotide content, with a guanine-rich strand referred to as the heavy strand (or H-strand) and a cytosine-rich strand referred to as the light strand (or L-strand) (28). The H-strand encodes 28 genes, and the light strand encodes 9 genes for a total of 37 genes (28). Of the 37 genes, 22 code for transfer RNA (tRNA), 2 code for small and large subunits of ribosomal RNA (rRNA), and 13 code for ETC proteins that are essential for cellular metabolism (28). Interestingly, the ETC is unique in that the complexes are composed of subunits encoded by two different genomes; the mitochondria and the nucleus (30). Thirteen of the mtDNA genes encode for proteins of complexes I (MT-ND1, MT-ND2, MT-ND3, MT-ND4, MT-ND4L, MT-ND5, MT-ND6), III (MT-CYB), IV (MT-CO1, MT-CO2, MT-CO3) and V (MT-ATP6, MT-ATP8), and the remainder of the ETC, of which there are over 80 genes, are encoded by the nucleus (30).

In contrast to nuclear DNA, which contains at least one promoter within a gene, mtDNA has only three promoters, heavy strand promoter 1 (HSP1), heavy strand promoter 2 (HSP2), and light strand promoter (LSP) (28, 29). The D-loop, a regulatory sequence that controls mtDNA replication and transcription, contains the HSP1, HSP2, and LSP. Transcription in the mitochondria is initiated bidirectionally from the HSPs and LSP (28, 29). Furthermore, the genes of the mitochondria lack introns, intergenic sequences, and many stop codons (28, 29). The gene sequence allows the translation of all codons using less than the usual 32 tRNA molecules because a single tRNA with U in the first anticodon position binds to all codons in a 4-codon family. Several proteins necessary for mtDNA replication and transcription include mitochondrial RNA polymerase (POLRMT), mitochondrial transcription factor A (TFAM), mitochondrial transcription factors B1 and B2 (TFB1M, TFB2M), and the transcription termination factor (mTERF), which are all nuclear-encoded (31). Taken collectively, mitochondria structure, function, and transcription rely on a strong interplay between the mitochondria and nuclear genomes. This mini-review is not intended to be a review of the crosstalk between mitochondria and nuclear genomes, and the reader is referred to several excellent reviews in this area (32–34).

### Mitochondrial abnormalities: Hallmark of skeletal muscle insulin resistance

It is well established that mitochondrial content, abundance, and bioenergetic function are lower in the insulin-resistant muscle of volunteers with T2D or obesity (19, 35–39). The work of Kelley et al. demonstrates a lower mitochondrial mass and decreased mitochondria surface area in the skeletal muscle of insulin-resistant individuals with and without T2D compared with healthy lean volunteers (19). They also showed that the overall capacity of the ETC, as measured by the specific activity of rotenone-sensitive NADH:O2 oxidoreductase, was reduced in human skeletal muscle from insulin-resistant volunteers (19). Additionally, multiple studies observed a decreased expression of nuclear-encoded mitochondrial genes for proteins involved in electron transport chain (ETC) and oxidative phosphorylation (OXPHOS) in insulin-resistant muscle, and these changes were attributed to lower peroxisome proliferator-activated receptor gamma coactivator 1 alpha (PPARGC1A) expression (40, 41). A study by our laboratory showed that experimental insulin resistance reduced PPARGC1A and other nuclear-encoded mitochondrial oxidative phosphorylation genes in skeletal muscle (42). Consistent with these studies, a global analysis of protein abundance changes in muscle biopsies from metabolically characterized lean and insulin-resistant patients demonstrated a reduction in ETC/OXPHOS proteins in naturally occurring insulin-resistant muscle (43). These data clearly demonstrate impaired mitochondria structure and function in insulin-resistant muscle. The lower abundance of mRNAs and proteins coding for the ETC in skeletal muscle of insulin-resistant volunteers may be caused by epigenetic regulation of these genes that are encoded by both mtDNA and nuclear DNA. The remainder of this review will describe epigenetics and discuss the current DNA methylation findings in the context of IR and mitochondrial abnormalities of both the nuclear and mitochondrial genomes.

### Epigenetics

Waddington initially coined the term ‘epigenetics’ to describe mechanisms of inheritance that operated with and above standard genetics (44, 45). According to Weinhold, the term has evolved throughout the years to mean any process that alters gene activity without changing the DNA sequence (46). The most commonly researched epigenetic mechanisms are histone modifications, microRNAs, and DNA methylation (46–50). There has been considerable research on nuclear genome epigenetic mechanisms, as described in detail elsewhere (46–50). Some of the complexities of studying epigenetics include the reversibility and inheritability of epigenetic patterns and that environmental factors such as diet and physical activity can have an effect (51–53).

Histones, which are known to form nucleosomes with nuclear DNA, but are absent from mitochondria, can be modified in several ways, including acetylation, phosphorylation, and methylation (47). Modifications to the histones can result in altering the gene’s activity. Histone acetyltransferase and histone deacetylase are post-transcriptional modifications that add or remove an acetyl group to the histone (54). These acetylation modifications alter the gene’s activity, for example, the loosening of the histone to increase replication or tightening of the histone to decrease replication (54). Additionally, modification of histones can occur through histone methyltransferases, which specifically add a methyl group to the arginine or lysine residues on the histones (55). The methylation or demethylation at the histones can either cause transcription activation or inhibition due to the loosening or tightening of the DNA structure (55). Histone modifications also include phosphorylation and ubiquitination. Histone phosphorylation is best known to take place during cellular response to DNA damage (56). Histone ubiquitination plays a role in transcriptional regulation and DNA repair by binding to highly conserved lysine residues on satellite regions of the genome and gene body of transcriptionally active genes (57). Importantly, these modifications can impact the accessibility of polymerases and transcription factors to access the DNA for gene expression regulation.

MicroRNAs (miRNAs) are non-coding RNAs approximately 18-25 nucleotides long (50). They act as epigenetic modulators in conjunction with DNA methylation, RNA modification, and histone modifications (50). They post-transcriptionally regulate gene expression by regulating target mRNAs. They do this by binding to the untranslated regions (UTRs) of mRNA in RNA interference (RNAi) machinery, which causes suppression or decay of the mRNA (58, 59).

DNA methylation is one of the most commonly studied epigenetic mechanisms, partly because the technology to measure methylation has significantly improved over the years. It is also a potential mechanism by which gene expression may be regulated (48). In nuclear genes, methylation commonly occurs on CpG dinucleotides in double-stranded DNA (48). DNA methyltransferase (DNMT) enzymes add a methyl group to the cytosine’s C-5 position (60). The most abundant enzyme from this group in somatic cells is DNMT1, which is heavily involved in maintaining methylation patterns between parent and daughter cells after replication (61). The C-5 position of cytosine represents 0.75-1% of all nucleotides, where 4-6% of all cytosines are methylated (62). In nuclear DNA, CpG dinucleotides concentrate together to form CpG islands (62), most commonly occurring in the promoters of nuclear-encoded genes (62). The amount of methylation at the gene’s promoter can impact transcription; for example, increased methylation levels at these CpG islands can cause gene silencing (48).

### DNA methylation: Emphasis on mitochondrial abnormalities in skeletal muscle IR

Various groups, including our own, have looked at the role of DNMTs in insulin resistance. In our study, we showed that the mRNA expression of DNMTs was unaffected by 8-weeks of exercise training in the skeletal muscle of volunteers with a range of insulin sensitivities (63). Moreover, a study in pancreatic islets from human donors with and without T2DM revealed no gene expression difference for DNMT1 and DNMT3B (64). In that same study, however, the authors showed a trend toward reduced DNMT3A expression in the T2DM islets compared with the non-T2DM islets (64). Furthermore, You et al. showed that adipose-specific Dnmt3a knockout mice are protected from diet-induced insulin resistance (65). Moreover, another study using human skeletal muscle cells in culture provided evidence that selective silencing of the DNMT3B, but not DNMT1 or DNMT3A, prevented palmitate-induced methylation of PPARGC1A and decreased mtDNA and PPARGC1A mRNA (66). These studies are only a few of the many that highlight the importance of DNMTs in metabolic diseases and its underlying insulin resistance. Targeting the DNMTs through pharmacological inhibition might have a beneficial effect on treating diseases such as diabetes and obesity, as discussed in the review by Arguelles et al. (67).

Several studies have demonstrated that the degree of DNA methylation in the nuclear-encoded ETC and OXPHOS genes cytochrome c oxidase polypeptide 7A1, NADH dehydrogenase 1 beta subcomplex subunit 6 and PPARGC1A correlated negatively with gene expression changes in insulin-resistant skeletal muscle (68–72). There are several outstanding reviews on this topic, and we recommend the following to the readers (73, 74). Recently, there has been a renewed interest in studying mitochondrial epigenetics, particularly DNA methylation, and determining the role that methylation of mtDNA may play in transcription. Therefore, the remainder of this review will focus on the DNA methylation of the mtDNA, emphasizing studies performed in insulin-resistant T2D or obese volunteers.

Studies investigating epigenetic regulation of mitochondria mtDNA have been lacking, partly due to the report over 45 years ago by Dawid, who showed a lack of evidence for mtDNA methylation (75). However, the field has been revived by the discovery of the nuclear-encoded DNMT1, which translocates to the mitochondria *via* a mitochondrial targeting sequence immediately upstream of this gene’s commonly accepted translational start site (76). In that same study, the authors demonstrate that the mitochondrial DNMT1 appears responsible for cytosine methylation of the mtDNA (76). In contrast, a recent study using both targeted- and whole mitochondrial genome bisulfite sequencing approaches showed that cytosine methylation is nearly absent in mtDNA (77).

There have been some key reviews that have described the existence of mtDNA methylation and its subsequent roles in disease. For example, a 2017 review provides evidence of mtDNA methylation; in particular, they discuss that mtDNA exhibits non-CpG methylation patterns similar to those found in prokaryotic genomes (78). Also, the same review described differential methylation of the mtDNA strands in and around the regulatory D-loop (78). Furthermore, a 2021 review described several studies demonstrating mtDNA methylation in animal and human tissues, especially in the D-loop region, and showed that mtDNA methylation could affect transcription (79). In that same review, the authors suggest that earlier studies looking at mtDNA methylation failed to find evidence as it occurs at much lower levels than nuclear DNA methylation (79).

We know that mitochondrial abnormalities (for example, lower abundance of ETC mRNAs and proteins) exist in the skeletal muscle of insulin-resistant volunteers and looked for evidence that methylation of the mtDNA may contribute (19, 35–42). Only a few studies have looked at mtDNA methylation changes in skeletal muscle. A recent paper showed that mtDNA methylation in the mitochondrial encoded cytochrome B gene increased in muscle tissue of myopathy patients compared to healthy controls (80). Furthermore, Wong et al., determined whether methylation occurred in mtDNA in skeletal muscle of mice with amyotrophic lateral sclerosis (ALS) (81). In that study, the mice with ALS had differential DNA methylation in the D-loop and 16S ribosomal gene (81). The study of Ruple et al. looked for changes in mtDNA methylation with resistance training in older male adults (82). The male adults had decreased methylation in the D-loop region following resistance training (82). Further, they showed enhanced expression of mitochondrial H- and L-strand genes and electron transport chain complex III/IV protein levels, suggesting that resistance training alters the mitochondrial methylome and function (82). These studies reveal that mtDNA methylation changes occur in skeletal muscle tissue, but they did not study insulin-resistant patients.

Although not in skeletal muscle, several studies have demonstrated the role of mtDNA methylation in metabolic diseases related to insulin-resistant states. A study using the bovine retina showed that exposure to high glucose levels correlated with increased mtDNA methylation, especially in the D-loop region, resulting in decreased mtDNA transcription, thereby leading to mitochondrial dysfunction (83). In that same study, the authors also found that retinal tissue from donors with diabetic retinopathy showed similar methylation patterns and mitochondrial dysfunction (83). In a study of type 2 diabetic rats who were obese and had impaired glucose tolerance, the severe retinopathy in these rats was due to lower mtDNA numbers and higher rates of mtDNA methylation (84). In addition, methylation of the liver mitochondrial encoded NADH dehydrogenase 6 (MT-ND6) associated with liver disease severity (85). A study in platelets from cardiovascular disease patients demonstrated significantly higher mtDNA methylation of the mitochondrial encoded cytochrome c oxidase I, II, and III (MT-CO1, MT-CO2, MT-CO3) compared to healthy controls (86). A more recent study of leukocytes from patients with acute coronary syndrome had significantly lower mtDNA copy numbers and higher levels of methylation in the D-loop region versus patients with stable coronary artery disease (87). The authors also suggest that the increased DNA methylation in the D-loop region translates into decreased mtDNA content and affects the clinical presentation of coronary artery disease (87). Moreover, another study showed that specific patterns of mtDNA methylation in platelets from overweight and obese individuals were strong predictors of future incidence of cardiovascular disease (88).

The results of several studies support the hypothesis that changes in mtDNA methylation correlate with insulin resistance and the subsequent conditions of prediabetes and T2D. A study using leukocytes from obese humans looked at the molecular mechanisms of insulin resistance; specifically, they set out to determine whether changes in the mtDNA may explain the mitochondrial abnormalities of insulin-resistant obese patients. They found reductions in mtDNA copy number and increased mtDNA D-loop methylation correlated with insulin resistance, which they propose was an insulin signaling-epigenetic-genetic axis in mitochondrial regulation (89). A different study measured fasting glucose, hemoglobin A1c, and mtDNA methylation levels to identify potential biomarkers of prediabetes (90). They showed that mtDNA methylation in the ND6 and D-loop regions was associated with early changes in insulin sensitivity (90). However, changes in A1c and fasting glucose levels did not correlate with any significant changes in the mtDNA epigenetics (90). A 2019 study used buccal swab samples to examine the connection between mtDNA methylation, mitochondria copy number, and body composition (91). The results showed a negative correlation between mtDNA copy number and BMI in females (but not in males), as well as significantly higher levels of D-loop methylation in overweight females than in lean females (91). Methylation of a specific CpG in the D-loop was associated with impaired body composition, and body weight appeared to be the best predicted by the combination of mtDNA copy number and D-loop methylation (91). Moreover, a 2020 study looked at mitochondrial methylation in vascular smooth muscle cells and showed that DNMT1 translocates to the mitochondria and induces D-loop methylation, which ultimately causes mitochondrial dysfunction (92). Taken collectively, these results indicate that methylation of the mtDNA should not be overlooked. Additional research needs to be performed in skeletal muscle tissue, particularly in human tissue, as it relates to the mitochondrial abnormalities that occur in T2D and obese volunteers with insulin resistance.

Lastly, insulin resistance and mitochondrial dysfunction are intertwined, and this review suggests that mitochondrial DNA methylation may play a role. This review does not fully determine whether the changes in mitochondrial DNA methylation are a consequence or a cause of skeletal muscle insulin resistance. Similar studies suggest that DNA methylation changes are a consequence of disease; for example, Wahl et al. showed that alterations in DNA methylation are the consequence of adiposity rather than the cause (93). Another study revealed that changes in DNA methylation are a consequence of T2D progression and highlighted the potential of utilizing methylation marks in the development of biomarkers for this disease (94). A review by Ling summarized data in the field that supported a causal role of methylation on impaired insulin secretion and action, which play significant roles in the pathophysiology of T2D (73). Future studies should focus on determining whether mitochondrial DNA methylation is a consequence or cause of skeletal muscle insulin resistance.

## Conclusions

Methylation of mtDNA is a rapidly growing area of research; however, more research needs to be performed to determine the mechanism of mtDNA methylation and discover whether the methylation is a cause or a consequence of disease. In addition, despite a large amount of research in this field, there is a lack of research on how mtDNA methylation relates to insulin resistance in skeletal muscle of obese and T2D volunteers. With evidence supporting the link between increased mtDNA methylation and both obesity and insulin resistance in other tissue types, more research needs to be performed on skeletal muscle.

## Author contributions

DC conceived the mini-review. ER, YH, and DC wrote the article. DC is the guarantor of this work and, as such, takes responsibility for the integrity of the review. ER, YH, and DC read and approved the final manuscript.

## Funding

This review was supported by the American Diabetes Association 1-19-ICTS-090 (DC).

## Conflict of interest

The authors declare that the research was conducted in the absence of any commercial or financial relationships that could be construed as a potential conflict of interest.

## Publisher’s note

All claims expressed in this article are solely those of the authors and do not necessarily represent those of their affiliated organizations, or those of the publisher, the editors and the reviewers. Any product that may be evaluated in this article, or claim that may be made by its manufacturer, is not guaranteed or endorsed by the publisher.
